# Pentosan Polysulfate Affords Pleotropic Protection to Multiple Cells and Tissues

**DOI:** 10.3390/ph16030437

**Published:** 2023-03-13

**Authors:** Margaret M. Smith, James Melrose

**Affiliations:** 1 Raymond Purves Laboratory, Institute of Bone and Joint Research, Kolling Institute of Medical Research, Faculty of Health and Science, University of Sydney at Royal North Shore Hospital, St. Leonards, NSW 2065, Australia; mobsmith@sydney.edu.au; 2 Graduate Schools of Biomedical Engineering, University of NSW, Sydney, NSW 2052, Australia; 3 Sydney Medical School, Northern Campus, Royal North Shore Hospital, St. Leonards, NSW 2065, Australia

**Keywords:** DMOAD, cystitis, anti-viral, tissue protection, hyaluronan, endothelial cell dysfunction, anti-inflammatory

## Abstract

Pentosan polysulfate (PPS), a small semi-synthetic highly sulfated heparan sulfate (HS)-like molecule, shares many of the interactive properties of HS. The aim of this review was to outline the potential of PPS as an interventional therapeutic protective agent in physiological processes affecting pathological tissues. PPS is a multifunctional molecule with diverse therapeutic actions against many disease processes. PPS has been used for decades in the treatment of interstitial cystitis and painful bowel disease, it has tissue-protective properties as a protease inhibitor in cartilage, tendon and IVD, and it has been used as a cell-directive component in bioscaffolds in tissue engineering applications. PPS regulates complement activation, coagulation, fibrinolysis and thrombocytopenia, and it promotes the synthesis of hyaluronan. Nerve growth factor production in osteocytes is inhibited by PPS, reducing bone pain in osteoarthritis and rheumatoid arthritis (OA/RA). PPS also removes fatty compounds from lipid-engorged subchondral blood vessels in OA/RA cartilage, reducing joint pain. PPS regulates cytokine and inflammatory mediator production and is also an anti-tumor agent that promotes the proliferation and differentiation of mesenchymal stem cells and the development of progenitor cell lineages that have proven to be useful in strategies designed to effect repair of the degenerate intervertebral disc (IVD) and OA cartilage. PPS stimulates proteoglycan synthesis by chondrocytes in the presence or absence of interleukin (IL)-1, and stimulates hyaluronan production by synoviocytes. PPS is thus a multifunctional tissue-protective molecule of potential therapeutic application for a diverse range of disease processes.

## 1. Introduction

**Figure 1:**
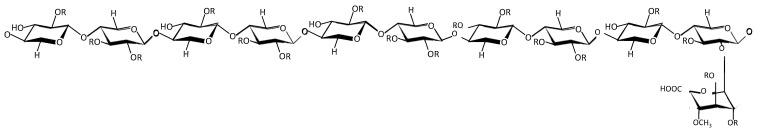
Structure of pentosan polysulfate showing its high sulfation density as proposed by Ghosh [6] and Ennemoser et al. [7].

Pentosan polysulfate (PPS) is a semi-synthetic sulfated xylan biomimetic heparinoid (Figure 1) that has been categorized as a disease-modifying anti-arthritic drug (DMOAD) and has been used clinically to treat cystitis, painful bowel disease and pelvic pain for decades [1,2,3,4]. PPS has a smaller molecular weight than heparan sulfate (HS) or heparin and has a higher charge density; however, it has many properties that mimic HS found on cell surface proteoglycans and in extracellular heparan sulfate proteoglycans (HS-PGs). This provides PPS with the ability to regulate physiological processes and equips it with multifunctional cell and tissue-protective properties. The aim of this review is to illustrate these multifunctional cell and tissue-protective properties of PPS and its subsequent usefulness as a therapeutic. PPS is a semi-synthetic xylan isolated from beech trees. Extracted xylan is treated with sulfating agents (e.g., chlorosulfonic acid or sulfuryl chloride), and after sulfation the PPS preparation is neutralized with sodium hydroxide to form a sodium salt of PPS [5].

## 2. Overview of PPS

PPS is reported to have lipid-lowering activity in tissues [8] and is commonly employed to treat cystitis, painful bowel disorder and pelvic pain; however, its precise mode of action has yet to be determined. It may improve barrier functions in affected tissues and it has antibacterial properties [4,9,10]. Oral PPS also blocks complement activation at similar concentrations to those used in heparin therapy (1–1000 µg/mL), indicating that PPS may have practical application as a complement inhibitor [11]. The retention of low-density lipoprotein (LDL) in blood vessels in plaque formations contributes to the pathogenesis of atherosclerosis and is largely mediated by smooth-muscle cell HS-proteoglycans such as perlecan [12]. Macrophages have also recently been shown to internalize lipids via complexation with perlecan [13], and perlecan also promotes lipid accumulation in adipocytes [14]. Perlecan clears lipids from the circulation through interactions between perlecan HS side chains in domain I and the LDLR-like motif in domain II [12,13]. The HS chains of perlecan inhibit smooth muscle cell proliferation, limiting the development of atherosclerotic plaques and development of atherosclerosis [15]. The HS chains of liver proteoglycans also clear lipids from the circulation, but independently of their core proteins [16]. As a heparin-like molecule, it is not surprising, therefore, that PPS should also display interactive properties with lipids [17]. This may explain the clearance of lipid-engorged subchondral blood vessels by PPS, which may significantly contribute to pain alleviation in OA [18]. HS is a well-known multifunctional glycosaminoglycan displaying a diverse range of bioactivities [19] and the pleiotropic properties of PPS mimic the diverse range of bioactivities displayed by HS. A recent publication proposed that the biotransformation of PPS by the gut microbiome converts PPS into health-promoting pre-biotics that may have a role in countering gut dysbiosis and the restoration and maintenance of a healthy gut environment [20]. The depletion of microbiota with immunomodulatory properties may prolong physiological effects on lung injury through the gut–lung axis [21]. Any delay in the re-establishment of these health-promoting gut symbionts may contribute to lingering effects induced by systemic inflammation [21,22,23].

Heparin inhibits SARS-CoV-2 attachment and infection in susceptible host cells [24]. PPS is as effective as unfractionated heparin, and more effective than low-molecular-weight heparin, in inhibiting viral infection [25]. When used at high therapeutic doses, heparin has serious side effects, including increased risk of bleeding, and its prolonged use can cause heparin-induced thrombocytopenia [26,27,28]. PPS is an alternative heparinoid therapeutic that has lower risk of these side effects since it exhibits weaker anticoagulant activity than heparin. Furthermore, PPS is well tolerated and has been used for decades to successfully treat interstitial cystitis and bladder pain without any reported toxic effects [1].

## 3. PPS Competes with and Mimics HS

PPS, as an HS-like molecule, shares many of the properties displayed by HS (Table 1). HS regulates a number of essential life-promoting physiological processes and directs cell behavior, not only in tissue development in embryogenesis but also in extracellular matrix (ECM) remodeling in repair processes following trauma and disease in mature musculoskeletal tissues. A recent study has illustrated the relevance of these HS interactive properties in tissue repair biology [29]. PPS has been used for decades in the treatment of cystitis and painful bowel disease [1,2,3,30,31,32,33], as a tissue-protective enzyme inhibitor [34,35,36,37], to promote cartilage, tendon and IVD remodeling [38,39], and in the repair of OA cartilage and the degenerate IVD [40,41,42,43,44,45,46]. PPS has also been used as a cell-directive component in bioscaffolds in tissue engineering applications [47,48,49], and to regulate complement activation [11,50], vascular coagulation, fibrinolysis [51,52,53,54,55] and thrombocytopenia [53,56], and in the regulation of HA production by some cell types [57,58]. PPS also inhibits nerve growth factor production in osteocytes, which reduces bone pain in OA/RA [59], and can remove excess lipid from subchondral blood vessels in OA/RA. This reduces pain in these conditions [45]. PPS modulates the differentiation and promotes the proliferation of bone-marrow-derived mesenchymal stem cells (MSCs) to progenitor cell lineages that have been employed in tissue repair strategies in the degenerate IVD and osteoarthritic articular cartilage [39,48,60,61]. PPS stimulates proteoglycan synthesis by bovine and ovine chondrocytes cultured in the presence or absence of IL-1, and also stimulates HA production by cultured RA and OA synoviocytes. HA has important tissue-protective properties [45,62,63,64,65,66].

### 3.1. PPS as a Therapy

Glycosaminoglycans have biodiverse tissue protective and regulatory properties which promote wound repair processes but are heterogeneous molecules that are difficult to consistently isolate in a pure form from tissues. This hampers their routine therapeutic application in the promotion of tissue repair following trauma and in disease. Historically, inadvertent co-purification of impurities in some therapeutic HS preparations resulted in serious unwanted side effects when these were used in clinical applications [87,88,89,90,91]. Since this troublesome contamination of heparin preparations was detected in 2008, methods have been developed to produce contaminant-free forms of heparin [87]. Procedures developed to reproducibly prepare PPS from beech wood hemicellulose overcome these difficulties and facilitate production of large quantities of PPS of well-defined purity. PPS is as efficient as heparin in potentiating the mitogenic activity of acidic fibroblast growth factor aFGF, acidic FGF) on human umbilical vein endothelial cells, it regulates cytokine and inflammatory mediator production [65,66,68] and it is an anti-tumor agent in a number of cancers [76,92,93]. PPS also protects against advanced glycation end-product-induced toxicity in diabetic nephropathy by inhibition of PI3K/AKT cell signaling [66]. PPS inhibits the activation of P38 MAPK cell signaling to protect against AGE-induced fibrosis, reduced cell proliferation, apoptosis and inflammation in the diabetic kidney, which results in renal diabetic nephropathy [65,68]. A reduction in the upregulation of proinflammatory genes in aging diabetic kidneys by PPS through suppression of NF-κB activity decreases the production of TNFα, IL-1β and IL-6, lowers inflammation, and protects against apoptotic effects in the aging kidney [65,68]. PPS binds FGFs and heparin-binding growth factors such as midkine (MDK) and pleiotropin (PTN) [94]. Midkine is a cancer biomarker [95,96]; PPS shows potential as an MDK cancer therapeutic [94,97,98], and targeting MDK abrogates IFN-γ-elicited metastasis in a number of cancer types [98]. PPS also interacts with the heparin-binding site of FGFR1 to promote FGF interactions that promote angiogenic events and wound repair processes [61]. Furthermore, PPS stimulates HA production by many cell types and this has well-known beneficial cell-directive properties, which promote cellular proliferation, ECM production and cellular migration, which contribute to reparative processes in tissues. PPS promotes FGF-signaling and actively engages in wound repair [61,80,99], and it has been widely used in veterinary practice as a DMOAD for the treatment of OA [81,100], preventing cartilage and tendon damage, and it promotes postoperative recovery of cartilage and tendon following instances of joint trauma [38,99,100]. PPS inhibits cartilage destruction in a virally-driven inflammatory OA model and reduces the inflammation that occurs during wound healing [73]. If left unchecked, inflammation can induce degenerative changes in joint tissues and pain.

### 3.2. The Role of Sulfation in PPS and Related Sulfated Molecules

**Figure 2:**
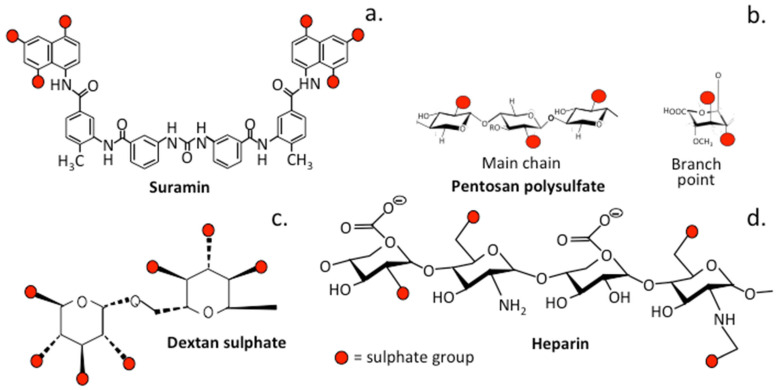
Structures of the sulfated glycopolymers suramin (**a**), PPS (**b**), dextran sulfate (**c**) and HS (**d**), showing their sulfation positions. See Figure 1 for details of an intact PPS chain.

Sulfate groups on a number of polymers have important cell-interactive properties and consequently have varied roles to play in cell-mediated processes that can be potentially harnessed in prospective therapeutic applications [101,102,103,104] (Figure 2). The extensive substitution of heparin, HS and PPS with sulfate groups outlined in this review provides important insights into the sulfation-mediated multifunctional cell-instructive properties of these compounds.

#### 3.2.1. Suramin

Suramin is a multi-ringed polysulfated multifunctional polymer that has been used for over 100 years to treat acute human sleeping sickness caused by *Trypanosoma bruceirhodesiense* [105]. Suramin has a wide range of potential applications; however, it also displays a number of minor side-effects, demonstrating its interactive properties with a number of organ systems, and it must be administered intravenously [105]. Human antigen R (HuR, human embryonic lethal abnormal vision-like protein) is an RNA-binding protein that regulates the stability, translation and nucleus-to-cytoplasm transport of target mRNAs, and it has emerged as an attractive cancer drug target. HuR is widely expressed by tumors, and suramin targets HuR [106,107] and inhibits HuR function to effectively suppress progressive oral cancer that cannot be treated using other anticancer agents [108]. Clinical trials have also been conducted with suramin for the treatment of prostate cancer [109]. Anti-metastasis suramin derivatives have also been prepared with superior anti-proliferative properties compared to unmodified suramin and these are also less toxic [110].

#### 3.2.2. Dextran Sulfate

Complement is an essential part of the innate immune system with crucial roles in organ and islet transplantation. Complement activation can significantly influence graft survival, and the blocking of complement by inhibitors has been shown to attenuate ischemia/reperfusion injury. Dextran sulfate can prevent activation of innate immunity, both in solid organ and islet transplantation [111]. Dextran sulfate binds to porcine endothelial cells and protects them from complement- and NK-cell-mediated injury in vitro, and it has been proposed as a novel therapeutic agent to prevent xenograft rejection [112]. Dextran sulfate also prevents Toll-like receptor induced maturation of human dendritic cells blocking the link between innate and adaptive immunity which can lead to rejection of transplanted tissue [113].

Suramin [114] and dextran sulfate are anti-tumor medications [115,116,117], displaying selective inhibition of K-FGF-induced tumor cell proliferation. Although heparin was inactive, PPS in contrast had a more than 2000-fold greater inhibitory effect on the growth of the human adrenal cancer cell line SW-13/K-fgf, as well as endothelial cells [118]. PPS also has inhibitory effects on tumorigenicity and metastasis of FGF-transfected MCF-7 cells [119]; thus, PPS was helpful in cases of breast carcinoma in which angiogenesis was due to the expression of FGFs by the tumor cells. PPS sequestered and controlled the bioavailability of growth factors, inhibiting tumor cell growth and resulting in diminution of the tumor mass [119].

### 3.3. PPS, Heparin and HS

PPS is a 4–6 kDa semi-synthetic sulfated beechwood xylan that is heavily sulfated and has a higher charge density and is less heterogeneous than heparin. PPS can be produced in a pure form, obviating the toxicity issues reported from heparin contaminants. PPS is a potent DMOAD [6,38,41] that has been used to treat cystitis and painful bowel disorder [1,120,121], and it has anti-viral and anti-tumor properties [92,93]. The backbone of PPS consists of β1-4 glycosidally linked xylopyranose residues, and one in every ten residues contains an *O*-2 linked 4-*O*-methylated *D*-GlcA side chain (Figure 1). PPS is heavily sulfated at O-2 and O-3. The O-3 sulfate group has a key role to play in heparin in the provision of its anticoagulant properties through the AT binding pentasaccharide; however, the O-3 sulfate moiety in PPS appears to convey less effective anticoagulant properties. PPS can inhibit thrombin and Factor Xa directly without relying on an intermediate such as AT to effect control over blood clotting.

Heparin is a polydisperse polysaccharide produced by basophils and mast cells that inhibits coagulation and occurs as a heterogeneous mixture of molecular weights ranging from 5–40 kDa in mucosal tissues [122]. Heparin is typically isolated from the lung and intestine; however, the HS fine structure in these tissues is highly variable. LMWH is prepared by size fractionation procedures or depolymerization of polymeric heparin. LMWHs have an average molecular weight <8 kDa with 60% of all chains being smaller than 8 kDa. LMWHs have lower risk of producing the osteoporosis or thrombocytopenia evident in non-fractionated heparin [123,124,125,126,127]. Fondaparinux, a synthetic pentasaccharide with a chemical structure almost identical to the antithrombin (AT) binding pentasaccharide sequence [128,129], was developed to target factor Xa rather than thrombin to provide a more subtle control of coagulation and a lower risk of thrombocytopenia [130,131,132,133,134]. PPS is also a relatively weak anticoagulant and does not have the same risk of thrombocytopenia side effects [52].

## 4. PPS and Coagulation

**Figure 3:**
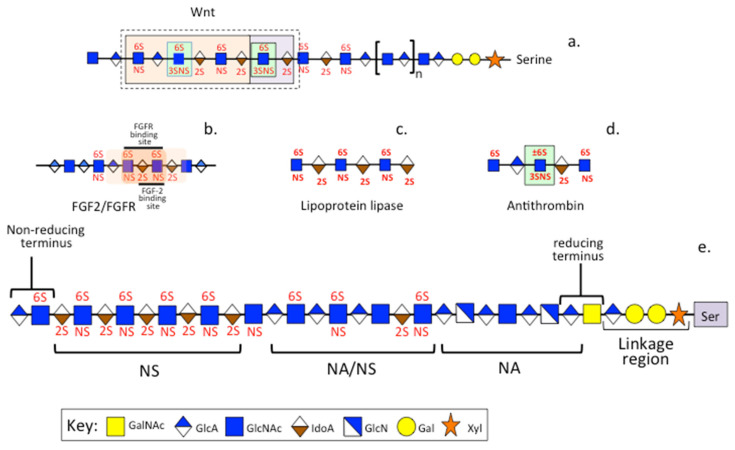
Binding sequences in HS determined for Wnt (**a**), lipoprotein lipase (**b**), antithrombin (**c**) and the FGF-2 and FGFR binding sites (**d**). Schematic depiction of the structural organization of a putative HS chain depicting its reducing and non-reducing termini, linkage region to a serine residue in a proteoglycan core protein. (**e**) Regions of high sulfation (NS domains), high acetylation (NA domains) and mixed regions (NA/NS domain) in a putative HS chain are also shown. Note: while heparin and HS have similar structures, heparin is fully modified and does not contain NA/NS domains, furthermore acetylation occurs in single units and not in block structures such as in HS. HS also is more heterogeneous in structure than heparin.

Upon binding of the heparin pentasaccharide to AT, a conformational change occurs resulting in AT activation, and an increase in AT inhibitory properties occurs with an increased inactivation of thrombin, Factor Xa and other coagulation cascade proteases up to 1000 fold [135,136]. Heparin binds to AT via the specific pentasaccharide sulfation sequence GlcNAc/NS(6S)-GlcA-GlcNS(3S,6S)-IdoA(2S)-GlcNS(6S) within polymeric heparin. Interaction of heparin with thrombin is an electrostatic interaction resulting in formation of an AT-thrombin-heparin ternary complex involving an 18 saccharide heparin segment [136]. Anti-Factor Xa activity, however, only requires the AT pentasacharide to effect inhibition. The heparin-like moiety HS also has interactive properties with a range of protease inhibitory proteins and induces conformational changes in these, which improves their inhibitory capacity and catalytic efficiency affording significant increases in their tissue-protective properties from excessive proteolytic degradation. HS is also a component of the extracellular HS-PG perlecan which has roles in matrix stabilization and acts as a co-receptor for the presentation of sequestered growth factors to a range of cells promoting cellular proliferation and differentiation in embryonic tissue development. Perlecan also promotes the vascularization and remodeling of connective tissues and has roles in tissue repair processes. Perlecan domain V also promotes repair of the blood–bone barrier following ischemic stroke and has been proposed to promote vascular repair processes. Thus, the HS component of perlecan has roles in tissue stabilization and cell and tissue protection [29]. The binding sequences in HS have been determined for a few of its ligands including Wnt, lipoprotein lipase, AT, and the FGF-2 and FGFR binding sites (Figure 3). These are also the most likely binding sites for PPS.

## 5. PPS and the Gut

Proteases are active in ulcerative colitis [137,138], colon cancer [139,140,141] and lung disease [142,143], and the resident protease inhibitory proteins have important tissue-protective roles [144], and in inflammatory bowel disease [145], which are boosted by interactions with heparin. Lung mucosal tissues also have a number of serpin protease inhibitors which afford tissue protection, and these include elafin, SLPI and α1-protease inhibitor. Heparin and HS enhance this tissue-protective effect; α1-protease inhibitor and members of the superfamily not only have tissue-protective roles in lung tissues but are also potent protective agents in the gut [143,146], and their interactions with heparin and HS further improve their protease inhibitory properties. A group of novel protease inhibitors, the siropins, are also supplied by the gut microbiome and these have tissue-protective properties [145,147]. A serpin from the gut bacterium *Bifidobacterium longum* inhibits elastase-like serine proteases [148] in a similar manner to PPS.

### 5.1. A Potential Health Promoting Role for PPS Processed by Gut Bacteria to a Pre-Biotic Xylo-Oligosaccharide

Xylans are an abundant hemicellulose of dietary terrestrial plants and seaweeds, and many of these form part of the human diet; however, the human genome does not contain enzymes capable of degrading this polymer. Xylans serve as non-digestible bulking dietary fiber that acts as roughage, improving the throughput of digested food material, lowering its transit time in the gut and improving gut health. The human gut microbiome contains members that can degrade xylans and also bio-transform PPS to xylo-oligosaccharides. Xylo-oligosaccharides are an “emerging” prebiotic that promotes the growth of beneficial bifidobacteria symbionts in the gut [149,150,151]. The gut microbiome has emerging roles in health promotion, communicating with a number of linked organ systems (e.g., lung, liver, brain) through the vagal nerve and makes important contributions to the health of these tissues [152,153]. This pre-biotic role for PPS is a novel feature for a therapeutic drug and further extends PPS’s therapeutic profile as a DMOAD anti-arthritic drug and prophylactic for the treatment of cystitis/painful bowel disease and pelvic pain [1,2,4,31].

### 5.2. Gut Content Transit Time and the Incidence of Bowel Cancer

A rapid transit of gut contents through the gastrointestinal tract has been suggested to minimize the development of colon cancers, with pre-biotic phytochemicals promoting the maintenance of a healthy bio-diverse symbiont community preventing the establishment of colonies of pathogenic organisms in the gut [154]. Besides the physical attributes of indigestible dietary fiber in the form of their water regain and their mucilaginous properties promoting a smooth throughput of digested material, some pre-biotic hemicelluloses contribute directly to this protective effect through cell-regulatory effects on the gut microbiome [155]; this may explain why small bowel tumors are relatively rare [156]. While therapeutic doses of PPS will not have enough mass to exert a bulking effect on the movement of gut contents, its bioconversion to xylo-oligosaccharide by the gut microbiome will nevertheless have a positive cell-directive effect.

## 6. The Chondroprotective Properties of PPS

PPS stimulates chondrocyte proliferation and differentiation [69], inducing ECM production in articular cartilage, and it stimulates HA production by synovial fibroblasts [157]. PPS is thus chondroprotective and there is a well-documented rationale for its use in the treatment of OA and RA [6]. Furthermore, PPS promotes proliferation and differentiation of bone marrow stromal mesenchymal stem cells to a chondrogenic phenotype [77,158]. PPS-stimulated chondroprogenitor stem cells have been used to treat OA cartilage [159]. PPS has recently been shown to inhibit NGF by osteocytes, reducing pain generation in OA joints. PPS also upregulates TIMP-3 production at the post-transcription level in cultured rheumatoid synovial fibroblasts and in the lining cells of rheumatoid synovium [35]. This is chondroprotective and counters the cartilage-degradative effects of the ADAMS and ADAMTS MMPs [36].

## 7. PPS and Stem Cells Used in the Repair of the Degenerate IVD

PPS promotes proliferation and differentiation of cultured stromal Mesenchymal stem cells (MSCs) isolated from bone marrow [77]. PPS acts as an HS mimetic to promote beneficial effects in the degenerate IVD and synergizes with co-administered MSCs to promote IVD repair processes [60,61]. PPS localizes in the nucleus of stromal stem cells, promotes development of chondroprogenitor cell lineages, ECM synthesis and discal repair by resident disc cells, offering new opportunities in discal repair biology [61]. In culture, PPS rapidly binds to MSC surface receptors, and is internalized and localized to the nucleus, inducing cell proliferation and differentiation, and facilitating expansion of chondroprogenitor cell lineages with improved proteoglycan synthesis and ECM synthesizing profiles [158]. Priming of MSCs with PPS enhances chondrogenesis and MSC proliferation by modifying basal gene and protein expression and offers a means of programming chondroprogenitor MSC lineages of potential application in the repair or regeneration of cartilaginous tissues in OA and degenerative disc disease [158]. In an ovine IVDD microdiscectomy model, 6 months after administration of MSCs, the disc proteoglycan (PG) content and matrix organization were improved in the lesion site relative to controls, representing a postsurgical adjunct limiting progression of disc degeneration after microdiscectomy [67]. MSCs and PPS combination therapy has been employed in tissue engineering in alginate and micromass culture methods to effect repair of degenerate IVD tissue [160]. MSCs have been used in combination with PPS in a microdiscectomy model of IVDD, embedded in a gelatin sponge sealed with fibrin glue in a microdiscectomy defect. This approach restored disc height, disc morphology and nucleus pulposus (NP) PG content [60]. MSCs and PPS have been used in PEG/HA-based hydrogels to treat IVDD [48]. When encapsulated in the hydrogels, MSCs retained good viability and rapidly adopted a rounded morphology, and the bound PPS in the hydrogel resulted in increased matrix formation when compared to the addition of soluble PPS to the hydrogel [47]. This injectable, degradable hydrogel, containing covalently bound-PPS and MSCs, has the potential to assist cartilage regeneration in IVDD. Co-administered PPS and MSCs have been used in a cervical model of IVD degeneration where a cage was inserted into an IVD defect and packed with a carrier containing MSCs alone or in combination with PPS to effect IVD repair [161]. Replacement of cartilaginous and bone tissue was observed as a reparative response in the degenerate IVD, and combination therapy was superior to MSCs used alone.

## 8. A Comparison of the Anticoagulant Properties of Heparin and PPS

**Figure 4:**
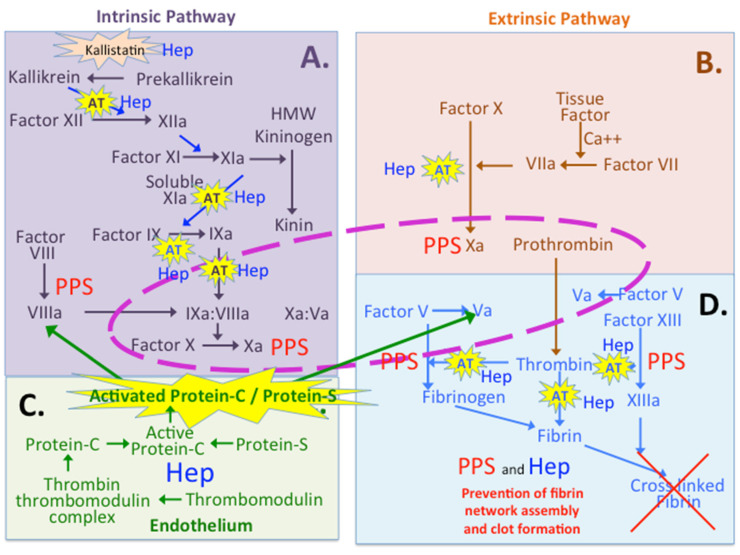
The coagulation cascade that regulates blood clot formation in wound repair and early containment of the wound site. The intrinsic pathway leads to sequential steps which generate Factor Xa (**A**). Factor Xa is also generated by the extrinsic pathway following tissue damage (**B**). Production of coagulation regulatory proteins in the endothelium (**C**). Thrombomodulin is a GAG substituted protein which has important roles in the regulation of the procoagulant proteins Protein C and Protein S. Thrombin and Factor Va have central roles in fibrin network assembly (**D**). The reactions surrounded by the central dotted area are those which occur on the platelet surface. Key steps in the generation of Xa, Va and VIIIa in the cascade are modulated by the heparin-regulated serine proteinase inhibitory protein AT in A and D. Kallistatin is another tissue serine proteinase inhibitory protein which regulates formation of kallikrein, which feeds into the coagulation cascade in early stages of the intrinsic pathway, and also regulates generation of kinins from kininogen which regulate vasodilation in early stages of wound repair. PPS inhibits thrombin directly in step D, preventing cleavage of fibrinogen and the creation of fibrin clot formation. Figure adapted from [102] with permission © Melrose 2016.

Heparin and PPS both exhibit anticoagulant activity by inactivating the coagulation proteases thrombin and Factor Xa [162,163] (Figure 4). Heparin has superior anticoagulant activity to PPS; however, PPS inactivates Factor Xa more effectively than heparin [162,163,164], and although the rate of inhibition of thrombin is lower for PPS, the rate of inactivation achieved is sufficient to elicit a physiologically significant response [165]. PPS inactivates thrombin via an AT-independent pathway [51,164].

### 8.1. Multiple Roles for Heparin and PPS in the Coagulation Cascades

PPS and heparin have multiple roles in the extrinsic and intrinsic arms of the coagulation pathways and in the endothelium (Figure 4). Heparin promotes the inhibitory activity of kallistatin, protein C inhibitor, HCII and AT [162,166,167,168]. PPS inhibits the generation of active Factor VIIIa and Xa and the activity of thrombin and Factor Xa [163,165,169].

Heparin/HS acts at several functional levels by (i) promoting kallistatin, PCI and AT inhibitory activities, (ii) inhibiting the generation of Factor VIIIa, (iii) inhibiting Factor Xa activity in the intrinsic and extrinsic pathways, (iv) inhibiting thrombin activity and (v) inhibiting cleavage of fibrinogen and fibrin network clot formation. Hep/HS induces a conformational change in shape in these inhibitory proteins that improves inhibitor binding kinetics to serine proteases and the capacity of these inhibitory proteins. PPS also inhibits the generation of Factor VIIIa and Xa and Factor Xa activity, and it inhibits thrombin directly without the requirement for AT in this inhibitory process.

### 8.2. Protein C Inhibitor and Thrombomodulin

Hemostasis, thrombosis and inflammation are tightly interconnected processes and platelets have central roles to play in these processes. Endothelial cell GAGs enhance the activities of the AT, tissue factor pathway-inhibitor (TFPI) and thrombomodulin-protein C systems [170]. Heparin/HS vasculo-protective heparin mimetics provide improved regulation of platelet and coagulation activity, suppressing production of endothelial and leukocyte-derived pro-inflammatory cytokines, and may be used to inhibit collagen-, thrombin-induced and complement-induced activation, providing organ protection from these potentially injurious processes [170]. Pro-coagulant, anti-coagulant and fibrinolytic pathways are responsible for maintaining hemostatic balance under physiological conditions. The Serpin superfamily are a complex mixture of protease inhibitors that have key regulatory roles in angiogenesis and coagulation. Binding of serpins to heparin and cell surface and ECM HSPGs have important regulatory roles over angiogenesis and coagulation, and potential in anti-coagulant and anti-angiogenesis therapeutic applications during inflammation, aiding in wound repair and tissue hemostasis [171]. Heparin acts through serine protease inhibitors such as heparin co-factor II, protein C inhibitor, tissue factor plasminogen inhibitor and AT in dynamic changes in remodeling connective tissues and in wound repair processes [172].

The endothelial-cell-dependent PC (protein C) pathway critically regulates coagulation, anti-inflammatory, and cytoprotective signaling [173] and is a major control system for thrombosis, and it limits inflammatory responses, and may also decrease endothelial cell apoptosis that may result from inflammatory cytokines and ischemia [174]. Essential components in this pathway include thrombin, thrombomodulin, endothelial cell protein C receptor (EPCR), protein C and protein S [174]. Thrombomodulin binding to thrombin directly inhibits its clotting and cell-activation potential and also augments protein C and thrombin-activatable fibrinolysis inhibitor (TAFI) activation. Thrombin bound to thrombomodulin is inactivated by plasma protease inhibitors > 20 times faster than free thrombin, resulting in an elevation in the clearance of thrombin from the circulation [174]; thrombomodulin is a bifunctional modulator of inflammation and coagulation [175]. Thrombomodulin’s epidermal growth factor-like domain 456 (TM456), enhances the catalytic efficiency of thrombin toward protein C and PCI by two to three orders of magnitude [176]. The major targets for PCI in blood are thrombin and activated protein C (APC); the inhibition of both enzymes is accelerated by interactions with glycosaminoglycans, including heparin [177]. Anionic phospholipids also accelerate the activation of PCI by Factor Xa; furthermore, the presence of PPS further improves this inhibitory process [178].

### 8.3. Kallistatin

Kallistatin found in plasma and tissues is a kallikrein inhibitor that has pleiotropic effects on angiogenesis, oxidative stress, inflammation and apoptosis, and it inhibits renal fibrosis and cardiac fibrosis following myocardial infarction and tumor growth [179,180,181,182,183,184,185]. Kallistatin levels are significantly reduced in coronary artery disease, sepsis, diabetic retinopathy, inflammatory bowel disease, pneumonia and cancer. Thus, interaction of HS with kallistatin to improve its inhibitory properties is of significant value in terms of tissue protection. Kallistatin antagonizes TNF-*α*-induced senescence, nuclear factor kappaB activation, and superoxide production, inhibiting oxidative stress and vascular injury in inflammatory conditions [180,186]. Kallistatin’s protective properties against the development of renal fibrosis is mediated through its ability to suppress TGF-β and β-catenin signaling pathways [180,182,186]. This modulates TGF-β-mediated fibroblast activation by Wnt4/β-catenin signaling, limiting epithelial to mesenchymal transition and fibroblast activation [185]. Kallistatin also suppresses cancer development through pleiomorphic effects [181]. Upregulation in the inhibitory performance of kallistatin through interaction with HS is yet another facet of how HS provides tissue protection. It will be interesting to ascertain to what extent PPS emulates these properties through interactions with kallistatin.

## 9. Heparin Inhibits Metastatic Events That Promote Cancer Development

MMPs, serine proteases and heparanases all have important roles in the metastatic process and cancer development. Heparin decreases the activation of these enzymes and limits their enzymatic effects [187]. Heparin and HS competitively inhibit tumor cell attachment to HS-PGs. Heparin and HS block the oncogenic effects of ornithine decarboxylase and enhance the antineoplastic properties of TGF-β [187,188]. Heparin and PPS inhibit AP-1, which is the nuclear target of many oncogenic signal transduction pathways [187]. Heparin blocks the phorbol ester-induced progression of non-transformed cells through the G0/G1 phase [189] or G1 to S phase of the cell cycle [190,191].

Cell cycle arrest occurs due to decreased levels of stage-specific mRNAs interrupting transcriptional regulation of cell growth. Heparin selectively represses TPA-inducible AP-1-mediated gene expression in primary VSMCs, a transformed HeLa cell line and in non-differentiated F9 teratocarcinoma cells. While heparin and PPS inhibit AP-1-mediated trans-activation, no effect is seen with CS-A or CS-C [191]. The oncogenic effects of PDGF are also inhibited by heparin [187,192]. The pro-oncogenic activities of reverse transcriptase, telomerase and topoisomerase are inhibited by heparin and HS [193]. Inhibition of ornithine decarboxylase by heparin and polyamine depletion has cytostatic effect on proliferating endothelial cells, and in combination with the anti-angiogenic properties of PPS through effects on the bioavailability of heparin-binding growth factors it could be a means of inhibiting tumor angiogenesis. The squamous cell carcinoma antigens (serpins B3 and B4) are tumor-associated proteins that can inhibit papain-like cysteine proteases, including cathepsins L, K and S. This is an example of glycosaminoglycan enhancement of B-clade serpin activity with heparin acting as a cofactor in serpin cross-class inhibition of cysteine proteases. This raises the possibility that the anticancer properties of heparin may be due, at least partly, to enhanced inhibition of pro-metastatic proteases [194]. HCII is a serpin whose thrombin inhibitory activity is also accelerated by glycosaminoglycans [195]. Accelerated thrombin action is associated with insulin resistance, and upon activation by binding to DS- and HS-, HCII rapidly inactivates thrombin in tissues. HCII regulates glucose homeostasis by regulating insulin sensitivity and methods that improve HCII production may be novel therapeutic tools for the treatment of type 2 diabetes. Thus, the improvement of HCII inhibitory activities and inhibitory capacity by sulfated polysaccharides needs to be examined further [196].

The relative potency of PPS for the activation of heparin cofactor-2 (HC-II)/thrombin or antithrombin/ thrombin interaction in comparison to heparin and dermatan sulfate was investigated and found to be of the same order [163]. It was possible to differentiate between high (~20 kDa), average (4.5 kDa) and low molecular weight fractions of PPS by their HC2 activity (high, low and none, respectively). PPS binding to antithrombin III and thrombin was a pre-requisite of activation. The control of coagulation by PPS was principally through inhibition of thrombin, mainly (>80%) compared to HC II.

## 10. Heparin and PPS in Bone Health

Long-term administration of unfractionated heparin can induce osteoporosis and is regulated by heparanase production by osteoblasts which lay down bone. HS is involved in osteogenesis via coordinating multiple signaling pathways. The potential effect of HS on osteogenesis is a complicated and delicate biological process, which involves the participation of osteocytes, chondrocytes, osteoblasts, osteoclasts and a variety of cytokines [197,198,199,200,201,202].

A major problem in modern health care is the emergence of oncological disease in the elderly and in the young. Inadequately effective chemotherapy is associated with the emergence of multidrug-resistant tumor cells. This is a major problem that can occur through immunosuppression mediated directly by the tumor cells themselves and also induced by antitumor drugs. The search for ways to overcome these pharmacologically resistant malignant cells is thus an important clinical objective. Heparin has broad biological activity, including roles in immunomodulation, and could potentially be focused to alleviate these antitumor effects [203]. Osteosarcoma is an example of a rare, highly aggressive, chemoresistant malignant tumor affecting young adults and characterized by recurrence and metastasis. The direct and immune-mediated regulatory effects of heparin on drug delivery systems in pathological bone tissue such as osteosarcoma is producing significant improvement in the treatment of these clinically difficult tissues [203]. Bone tumors are difficult to treat, with the efficacy of the therapeutic agents being compromised by the physiological bone environment. Alendronate (ALN) and low molecular weight heparin (LMWH) modified liposomes have been used to deliver the antitumor drug doxorubicin (DOX) [204]. ALN, rather than acting as an inert drug carrier, serves as a bone-targeting component with anti-osteoporotic properties. LMWH in this formulation enhances blood circulation and the therapeutic window for liposome delivery of DOX to tumor cells, improving anti-metastasis efficiency. Results with this delivery system have provided remarkable suppression of tumor growth and also significant inhibition of tumor metastasis [204]. A DOX/LMWH nano-particle chemotherapeutic delivery system has also been developed to treat breast cancer [205]. Moreover, the anti-tumor efficiency of DOX was enhanced and its toxicity lowered, and the bleeding effects of LMWH were eliminated, by using this nano-particle approach to drug delivery [205]. These nano-particles had extended blood circulation transit-time and exhibited a rapid triggered release of DOX at the tumor site. The nano-particles also inhibited cell migration and invasion, and the formation of tube-like structures by human umbilical vein endothelial cells, and they exhibited greater reduction in tumor mass compared to lipoic acid unmodified nanoparticles and free DOX [205]. These DOX nano-particles exerted antitumor, anti-metastasis and anti-angiogenesis efficacy simultaneously and had lower systemic toxicity in the treatment of metastatic breast carcinoma. Nano-particles have also been prepared using DOX, ALL-trans retinoic acid and LMWH (DOX-ALLT-LMWH) [206]. These DOX-ALLT-LMWH nano-particles were efficiently taken up by tumor cells by endocytosis, and initially stored in the cytoplasm, then transported to the nucleus. The DOX-ALLT-LMWH nano-particles possessed much higher anticancer activity and reduced side effects compared to free drug solutions [206]. These results suggested that DOX-ALLT-LMWH could be considered as a promising targeted delivery system for combination cancer chemotherapy with lower adverse effects [206]. LMWH has been advocated for micro- and nano-particle delivery of drugs [207]. PPS has similar properties to LMWH in many cellular systems and in vitro studies, and thus it will be interesting to ascertain if PPS can also be used in such nano-particle chemotherapeutic delivery systems.

Heparin-binding growth factors are essential for the maintenance of a blood supply to enable tumor growth in vivo. PPS acts by blocking the paracrine effects of heparin-binding growth factors released from the tumor cells and represents a novel tool for the specific targeting of tumor growth factors [93]. When tumors attain a few millimeters in size, a neoangiogenic response is critical to their unrestricted growth through release of angiogenic growth factors by tumor cells. This stimulates blood vessel growth to maintain the nutrition of the tumor mass. Blockade of such stimulatory activity represses tumor growth [118]. A human adrenal cancer cell line (SW-13/K-fgf) engineered to secrete Kaposi’s sarcoma-derived fibroblast growth factor (K-FGF), has shown that growth of highly vascularized subcutaneous tumors occurs in animals through autocrine and paracrine stimulatory factors [118]. PPS inhibits these angiogenic processes.

PPS is a promising therapeutic agent for blocking pain in individuals with knee OA [59]. NGF/proNGF are produced by osteocytes in knee OA and NGF is also implicated in the pain response in OA. PPS inhibits TNFα-induced proNGF secretion and TNFα-induced NGF mRNA expression. PPS suppresses the release of NGF in the subchondral bone, ameliorating the pain associated with knee OA [59]. Currently, there are no registered products for the treatment of subchondral bone marrow edema lesions and associated knee pain. Treatment of subchondral bone marrow edema lesions in adult advanced OA patients with calcium phosphate is ineffective [208]. The effect of intramuscular PPS injections twice weekly for 3 weeks was examined in one patient with the treated knees evaluated by MRI [45]. There appeared to be complete resolution of the bone marrow edema in the medial femoral condyle and medial tibial plateau with concomitant recovery from pain 2 weeks post-treatment. Further studies into the efficacy of PPS in the treatment of bone marrow edema lesions and associated pain in OA are ongoing [45].

Hepcidin is a crucial master regulator of iron homeostasis produced in the liver in response to anemia, hypoxia or inflammation. Hepcidin and iron metabolism have recently been shown to have roles in osteoporosis by inhibiting osteoblast function and promoting osteoclastogenesis [209]. PPS down-regulates osteoclast gene expression, including cathepsin K and MMP-9, which have roles in bone metabolism. PPS also inhibits osteoclast differentiation and proliferation [209,210]. PPS reduces serum and tissue levels of TNFα, MIP-1α and RANTES/CCL5 in Mucopolysaccharidosis (MPS) type VI rats [211]. PPS could be a simple and effective therapy for MPS that might provide significant clinical benefits when used in isolation and in combination therapies. PPS also produces a significant reduction in inflammatory cytokine production by cultured articular chondrocytes, reduced expression of inflammatory markers [68,69] and reduced ADAMTS-5/aggrecanase-2 levels [36,37,68,69].

## 11. Regulation of Hepcidin and Iron Metabolism by PPS in Chronic Diseases

Hepcidin, a peptide hormone, acts as a central regulator of iron metabolism, regulating the uptake of dietary iron and its mobilization from macrophages and hepatic stores, and it is considered as a mediator of anemia of inflammation. Serum prohepcidin is clearly reduced in uncomplicated iron deficiency anemia. Reduced prohepcidin levels also occur in iron-depleted RA patients [212,213,214]. Rheumatoid anemia is a typical example of anemia in a chronic disease and may also be accompanied by osteoporosis and thrombocytosis [215,216]. Hepcidin is a peptide hormone that lowers serum iron levels regulating iron transport across membranes, preventing iron from exiting the enterocytes, macrophages and hepatocytes. Hepcidin inhibits absorption of iron from the intestine and the release of iron from macrophages and hepatocytes. This action of hepcidin is mediated by binding to the iron exporter protein ferroportin. Oncology studies and studies evaluating the effects of recombinant human IL-6 support a causal link between IL-6 production and the development of anemia in patients with chronic disease, contributing to inflammatory conditions in chronic-diseased tissues [214]. Hepcidin is upregulated in renal cell carcinoma and associated with poor survival rates [217]. Iron overload is also associated with the upregulation of hepcidin in polycystic ovary syndrome [218]. Iron restriction has been shown to alleviate atherosclerosis in ApoE knock-out mice [219]. Inhibition of hepcidin-induced osteoclast proliferation and function by PPS may be useful in the treatment of OA and osteoporosis and in the promotion of bone health [210,220]. Once weekly intramuscular injections of 3 mg/kg PPS given to Mongolian horses for four weeks before racing reduced serum hepcidin after racing when compared to placebo injections [144]. Control of hepcidin is thus an important cell and tissue-protective action of PPS.

## 12. PPS as an Anti-Tumor Agent in a Model of Gastric Cancer

The biological phenotypes of gastric cancer cell lines are regulated by MK, a novel heparin-binding growth/differentiation factor. PPS inhibits the growth of MK-expressing cells. In cancer tissues, MK expression correlates with tumor size; this proliferation-promoting activity of MK has been targeted by PPS as an anti-heparin binding biotherapeutic agent [94].

Combination therapy of PPS and adriamycin have also been examined in an Adriamycin-resistant MK-expressing human gastric cancer cell-line (YCC-7) [94,221]. PPS suppressed the colony-forming properties of the YCC-7 cell line and also displayed cytostatic effects on cell proliferation. Growth inhibition was inhibited 84%, apparently through PPS’s action as a heparin-binding growth factor inhibitor, limiting the bioavailability of growth factor which inhibited tumor progression [94,221].

## 13. Beneficial Combination PPS-MSC Therapy in a Model of Interstitial Cystitis

Combination therapy with PPS and adipose-tissue-derived MSCs showed histological and functional effects in an interstitial cystitis rat model, including increased intercontraction interval, reduced pain scores and decreased inflammatory reactions. Histological analysis revealed regenerated urothelium, less fibrosis and decreased mast cell infiltration, and significantly lower expression of TNF-α, IFN-γ, MCP, IL-6, TLR2 and TLR11 [222].

## 14. Anti-Viral Properties of PPS

PPS has anti-viral activity against all categories of virus, whether they be negative single-strand RNA-, positive single-strand RNA- (like coronaviruses) or double-stranded DNA forms. A flow-cytometry-based method to measure inhibition of viral infectivity demonstrated the antiviral activity of PPS, suramin and PI-88 in vitro. The 50% effective concentration (EC50) values for dengue virus (DENV) inhibition were in the order PPS < suramin < PI-88, and for Japanese encephalitis virus (JEV) they were PPS < PI-88 ≤ suramin. However, the in vitro anti-flaviviral effectiveness of these polysulfates did not reliably predict their in vivo therapeutic activity in mice [223]. PPS is a selective, potent and non-cytotoxic inhibitor of Junin virus (JUNV) and Tacaribe virus (TACV), both negative single-strand RNA arenaviruses [224]. PPS and dextran sulfate inhibited pathology by HIV (+ssRNA virus) and three types of HSV and HCMV (dsDNA viruses) [225]. PPS also inhibited herpes simplex virus (HSV) infectivity and the cell-to-cell viral spread [226,227]. In vitro, PPS inhibited HSV-1 adsorption to green monkey kidney (GMK) cells if added with the virus but not if the cells were pre-incubated with PPS [228].

Like the coronaviruses, HIV is an enveloped +ssRNA virus. PPS and several other sulfated molecules such as fucoidan, dextran sulfate and heparin are potent and selective inhibitors of human immunodeficiency virus type 1 (HIV-1) in vitro [70,229]; however, PPS was the most potent anti-HIV-1 agent with a 50% antiviral effective dose (ED50) of 0.19 μg/mL in MT-4 cells, with no toxicity observed. It completely inhibited HIV-1 antigen expression in HuT-78 cells at 4.0 μg PPS/mL. No toxicity for MT-4 cells was observed even at 2500 μg PPS/mL. PPS achieved its anti-HIV-1 activity at a concentration 370-fold below its anticoagulant threshold. The inhibitory effect was dose-dependent and reversible [230], with no cell–cell fusion (syncytium formation) or destruction of bystander T cells observed [231]. PPS inhibited HIV-1 virus adsorption to MT-4 cells [70], CD4+ HeLa cells [232] and a human T-cell line (8E5) [233]. More specifically, the negatively-charged polysulfated polysaccharides shield the positively-charged amino acid residues in the V3 loop of the viral envelope glycoprotein gp120 with the inhibition by PPS being CD4 independent [234,235,236,237,238]. PPS was able to block the CD4-gp20 interaction in lymphocytes, but not in monocytes, however [239]. HIV-1 attachment to HeLa cells was only partially inhibited by monoclonal antibodies specific for adhesion molecules present on the virus or target cells, but was completely blocked by PPS [240]. Treatment of HeLa-CD4 cells with heparanases completely eliminated HIV-1 attachment and infection, confirming the role of cell-surface heparan sulfates in the attachment process. The inhibition of Tat activity also contributes to the anti-HIV-1 action of PPS (and other sulphated polysaccharides) [63,241]. This HIV regulatory gene is essential for viral replication; the Tat protein is released from virally infected cells, enters new cells in an active form and stimulates transcriptional activity of HIV [242]. PPS exerts its Tat antagonist activity with an ID50 equal to ~1.0 nM. In vivo, PPS inhibits the neovascularization induced by glutathione-S transferase-Tat or by Tat-overexpressing T53 cells in the chick embryo chorioallantoic membrane [63].

Another mechanism whereby PPS exerts its inhibitory effect on HIV-1 replication is by inhibiting various enzymes necessary for the process, such as the protein serine/threonine and tyrosine kinases. The inhibition of rat lung protein tyrosine kinase was rapid and competitive with respect to ATP with an apparent Ki value of 5–20/µg/mL. PPS also inhibited lymphocyte protein tyrosine kinase, human platelet protein kinase C and the catalytic subunit of cAMP-dependent protein kinase from skeletal muscle [76]. PPS was also a potent inhibitor of HIV-1 hybrid-degrading RNase H activity in vitro, with an IC50 = 0.04–0.1 µg/mL [243]. This was 5000-fold more potent inhibition than that of the reverse transcriptase RNAase RT.

Human cytomegalovirus (HCMV) is an enveloped double-stranded DNA virus. PPS and other sulfated, natural or unnatural polysaccharides (e.g., dextran sulfate, heparin, copolymers of acrylic acid) can inhibit HCMV infectivity in CHO-K1 and MRC-5 cells [226,244] by mimicking the polymeric scaffold of heparan sulfate, which has now been shown to be critical for HCMV entry [245,246,247,248]. The most likely molecular basis for this competitive inhibition was the interaction of sulfated polymers with viral glycoprotein-gB of HCMV [249,250]. More specifically, the competitive inhibition arose by mimicking the structure of certain heparan sulfates, e.g., 3-O sulfated and 6-O sulfated species [247], and thus preventing the virus from interacting with these species on the host cells [244,251]. PPS at relevant concentrations inhibited infectivity of bovine viral diarrhea virus (BVDV) with permissive CTe cells [252] and the adsorption of African swine fever virus (ASFV) to Vero cells with low toxicity [71]. PPS can competitively inhibit binding of viruses to heparan-sulfate-proteoglycan (HS-PG) receptors. Inhibition of virus replication in CHO-K1 cells by PPS suggested that the coxsackievirus B3 (CVB3) was using a modified heparan sulfate for cell entry [253]. PPS inhibited adsorption of sandfly fever Sicilian virus (SFSV) to isolated Vero cells, causing a concentration-dependent reduction in viral yield [254]. PPS (and other heparan sulfate mimetics) also hindered attachment of enterovirus 71 (EV71) to, and its replication in, isolated Vero cells [255]. PPS significantly inhibited infection and attachment of Coxsackievirus A16 (CVA16) but not CVB4 to Vero and human neural cells [256].

The antiviral activity of PPS against human herpes virus type 7 (HHV-7) was also due to the blocking of different cell receptors, in this case, the CD4 receptor on T-lymphocytes [257]. Similarly, inhibition of dengue virus (DENV) by PPS was suggested as steric hindrance of virus attachment; however, the receptor for this virus is unknown [258].

PPS was ineffective at inhibiting influenza virus entry to MDCK cells [259]; however, fusion experiments (virus to cell membranes) with different influenza subtypes (H1N1 and H3N2) demonstrated that dextran sulfate (8 and 500 kDa) and PPS strongly inhibit the fusion activity as well as the in vitro replication of the influenza virus, with a good correlation between the two results [260,261]. A drug repurposing and biomarker identification preprint study, using comprehensive gene–disease associations through protein-protein interaction network analysis, identified PPS as a possible FDA-approved drug to repurpose for COVID-19 based on this binding capability [262], whereas another review suggested PPS as a broad spectrum anti-viral for possible use against SARS-CoV-2 [263]. Yet another study used molecular dynamics and consensus virtual screening and chose PPS as one of 35 from 8700 drugs as potential therapeutics that could be repurposed for COVID-19. PPS inhibited both attachment and infection of Vero cells by SARS-CoV-2 in vitro [7,24,264,265].

HTLV-1 was the first human retrovirus to be identified and is now endemic in certain areas worldwide [266]. HTLV-1 infection leads to inflammatory diseases such as polymyositis, dermatitis and tropical spastic paraparesis/HTLV-1-associated myelopathy (HAM/TSP), a myelopathy with slowly progressive spastic paraparesis [267]. PPS was investigated for its effects on HTLV-1-infected cells to provide evidence for its efficacy in HAM/TSP [74]. PPS was cytotoxic to certain HTLV-1-infected cells and significantly suppressed HTLV-1 virion production. PPS also efficiently inhibited HTLV-1 cell–cell transmission in T cells. In addition, PPS blocked HTLV-1 infection of primary endothelial cells (human umbilical vascular endothelial cells) and suppressed the subsequent induction of proinflammatory cytokine expression. Furthermore, PPS was found to inhibit the adhesion and transmigration of HTLV-1-infected cells. There is thus mechanistic evidence for the reported efficacy of PPS in a HAM/TSP clinical trial [268].

Visna virus is another retrovirus that causes inflammation of the central nervous system in sheep, leading to a slowly progressing neurological disease. PPS inhibited viral adsorption and fusion to ovine choroid plexus cells in culture without toxicity; however, it was 40-fold less effective than against the HIV-1 virus in MT-4 cells [269]. The fact that PPS and other polysulfated polysaccharides are inhibitory to some myxoviruses and retroviruses but not others may well depend on the amino acid sequences of the viral envelope glycoproteins involved in virus–cell binding and fusion [270]. Those viruses that are sensitive share a tripeptide segment (Phe-Leu-Gly), which may be a direct target sequence.

### 14.1. Animal Studies Demonstrating PPS as an Antiviral

Mouse models of Ross River virus (RRV) and CHIKV disease have been used to characterize the extent of cartilage damage in infection and investigate the potential of PPS to treat disease [72]. PPS treatment in mice infected with either virus significantly increased the anti-inflammatory cytokine interleukin-10 and reduced proinflammatory cytokines that are typically correlated with disease severity. The severe RRV-induced joint pathology, including thinning of articular cartilage and loss of proteoglycans in the cartilage matrix, was diminished with treatment, with PPS reducing the inflammation and joint swelling severity of both RRV- and CHIKV-induced arthritic disease [72]. In the CHIKV-infected mice, the functional decline was prevented by modulation of growth factor signaling and lymphocyte activation. PPS treatment led to a systemic reduction of the chemokines CXCL1, CCL2 (MCP-1), CCL7 (MCP-3) and CCL12 (MCP-5), all of which are involved with the reduction in cellular infiltrates, less inflammation and less joint swelling [271].

The anti-HTLV-1 effect of PPS in vivo was demonstrated using two transgenic mouse models [74]. PPS (100 ug/mL added to the cells before i.p. injection) blocked HTLV-1 infection in a mouse model with peripheral blood mononuclear cell (PBMC)-humanized NOD-scid IL2Rgamma null (hPBMC NSG) mice. PPS also suppressed the development of dermatitis and lung damage in HTLV-1 bZIP factor (HBZ)-transgenic (HBZ-Tg) mice, an HTLV-1 transgenic mouse model in which the mice develop systemic inflammation.

### 14.2. Clinical Trials of PPS as an Antiviral

A clinical trial was designed to test the effect of subcutaneous administration of PPS in 12 patients with human T lymphotropic virus type I (HTLV-I)-associated myelopathy/tropical spastic paraparesis (HAM/TSP) characterized by lower extremity motor dysfunction in an open-labelled design [268]. Subcutaneous PPS weekly (25 mg in week 1, 50 mg in week 2 and 100 mg in weeks 3–8) caused a marked improvement in lower extremity motor function, based on reduced spasticity, such as a reduced time required for walking 10 m and descending a flight of stairs. There were no significant changes in HTLV-I pro-viral copy numbers in peripheral blood, contrary to the inhibitory effect of PPS in vitro for intercellular spread of HTLV-1. However, serum soluble VCAM-1 was significantly increased without significant changes in the serum level of chemokines (CXCL10 and MCP-1). There was a positive correlation between increased sVCAM-1 and reduced time required for walking 10 m. PPS might induce neurological improvement by inhibition of chronic inflammation in the spinal cord, through blocking the adhesion cascade by increasing serum sVCAM-1, in addition to rheological improvement of the microcirculation. PPS has the potential to be a new therapeutic tool for HAM/TSP [268].

A phase I clinical trial of PPS (infused, subcutaneous or intralesion) in 16 patients with HIV-associated Kaposi’s sarcoma (AIDS-KS) was performed and PPS found to be well tolerated. In this small trial, no objective tumor response or evidence of anti-HIV activity was noted [75]. When PPS is given in conjunction with Zidovidine (AZT), lower doses of the latter can be used for effective HIV inhibition; this is a useful finding as AZT has several side effects, including anemia, neutropenia, mitochondrial myopathy and the development of resistance [272,273].

In a phase II clinical trial, PPS was administered to 16 patients with histopathologically confirmed AIDS-KS at the dose of 25 mg/m^2^ every 6 h on day one, followed by 25 mg/m^2^ every 12 h daily, by a subcutaneous injection. Patients were all males, median age 35 (27–43) years. A median of 5 (3–11) weeks of therapy was administered. Pain at the injection site and low-grade fever were the only toxicities observed. Drug-related effects on coagulation parameters or thrombocytopenia were not observed in the trial. There thus appeared to be objective antitumor activity with PPS in these AIDS-KS patients [92].

The safety and efficacy of subcutaneously injected PPS in 20 individuals with RRV-induced arthralgia was evaluated in a small double-blind placebo-controlled trial [73]. PPS or isotonic saline was injected twice weekly for 6 weeks and the drug was well-tolerated with overall joint symptoms showing near remission in 61.5% of PPS subjects compared to 14.3% of placebo subjects. Dominant hand grip strength was significantly stronger than placebo at day 15 (*p* = 0.019), and serum COMP (*p* = 0.049) and urine CTXII were reduced (*p* = 0.017).

HS has roles in viral entry steps during host cell infection, including the initial virus–receptor interaction, internalization, intracellular vesicular transport of viral components, and genomic release and transport of viral material into the host cell nucleus and viral replication. The development of live cell imaging methodology [274] offers a powerful tool for studying such dynamic viral cell entry infective events [275]. Development of advanced computer software to analyze viral in silico molecular docking interactions with prospective therapeutic molecules has also significantly aided in the identification of prospective anti-viral compounds that may be used to inhibit viral attachment to host cells and thus prevent viral infection. The efficacy of polyanionic SARS-CoV-2 anti-viral sulfated and phosphorylated polymers [7,276,277,278,279] as agents that can disrupt viral interactions with prospective host cell receptors is mediated through electrostatic interactions [265]. Phosphorylated polymers are an interesting development since these can be used to manufacture medical accessories with viral resistant surfaces [276,278,280]. Table 2 summarises the sulfated polysaccharides, including PPS which display anti-viral properties.

## 15. Conclusions

This review has shown that HS, heparin and PPS display biodiverse interactivities with many ligands that facilitate tissue development, ECM remodeling, wound repair and the regulation of essential cellular physiological life processes. The heterogeneity in heparin/HS structure may lead to unwanted side effects in pathological processes. In contrast, PPS has a defined structure, and it can behave like a heparin/HS-like molecule, but it does not display unwanted side-effects, making it a useful multifunctional therapeutic agent. PPS is a semi-synthetic sulfated xylan that can be produced consistently in high purity. To date, PPS has mainly found clinical application in the treatment of cystitis and painful bowel disorders; however, this review shows that it has beneficial properties in many other tissue processes such as chondroprotection, potential sports medical applications in joint tissue repair, stimulation of stem cell proliferation and differentiation of stem cell lineages of potential application in repair biology, as well as anti-viral, anti-bacterial and anti-tumor properties. PPS thus shows significant potential for the development of novel therapeutics in these areas of biomedicine. The potential PPS therapeutic applications are summarized in Table 3. Hopefully, this review will stimulate further therapeutic developments with PPS. Despite its relatively simple structure, PPS is capable of influencing a wide range of biological processes and has diverse tissue-protective properties.

## Figures and Tables

**Table 1:** Illustrative Examples of The Cell and Tissue Protective Properties of PPS.

Property	Refs
Treatment of cystitis, painful bladder syndrome, chronic pelvic pain	[1,2,3,31]
Promotion of repair of the degenerate intervertebral disc	[61,67]
Regulation of Complement Activation	[50]
Modulation of vascular coagulation, fibrinolysis and thrombocytopenia	[11,50,53,55,56]
Stimulation of hyaluronan synthesis by synoviocytes, fibroblasts and chondrocytes	[57,58]
Inhibition of NGF production by osteocytes providing pain relief in OA/RA	[59]
Lipid removal from engorged subchondral blood vessels in OA/RA and pain alleviation	[8,15,17,45]
Regulation of cytokine and inflammatory mediator production in ARDS	[65,66,68,69]
Disrupts cell surface viral HS interactions, prevents host cell infection/viral replication	[65,66,68,70,71,72,73,74]
Anti-tumor agent in many cancer types	[62,75,76]
Promotes BM stromal MSC differentiation/proliferation/expansion in tissue repair progenitor cell lineages	[61,67,77]
Tissue protective protease inhibitor. Multifaceted exosite inhibitor of aggrecanases, inhibits ADAMTS4 in OA chondrocytes, improves inhibitory properties of TIMP-3. Inhibits IGFBP- 5 proteolysis in articular cartilage in OA preserving IGF-I and II levels, cartilage integrity and functional properties	[37,78,79]
Protects cartilage from degradation in tendon transection models of joint destabilization that induce OA	[41,42,44,80,81]
Cartilage protective effects of PPS arise from its stimulation of proteoglycan synthesis by chondrocytes cultured in the presence or absence of IL-1, and stimulation of HA synthesis by RA and OA synoviocytes. HA also has cell protective properties in the glycocalyx	[38,69]
PPS inhibits IL-1β-induced iNOS, c-Jun and HIF-1α upregulation in canine articular chondrocytes in OA models	[82]
Inhibitor of extracellular HIV-1 Tat (trans-activator of transcription)	[63,83]
Improves cardiac function and tissue protection from action of ADAMTS4	[84]
Tissue protective properties in tendon PPS is a potent inhibitor of human granulocyte elastase, cathepsin B, cathepsin G, testicular and arterial hyaluronidase, N-acetylglucosaminidase	[85]
Protection of brain endothelial cells from damage by bacterial LPS-induced neuroinflammation	[64]
PPS inhibits inflammation and impedes progression of severe diabetic nephropathy	[65]
Amelioration of tissue fibrosis and inflammation through suppression of PI3K/AKT cell signaling	[66]
Decreases prostate smooth muscle cell proliferation and ECM production	[62]
PPS has tissue protective properties in chronic non-bacterial prostatitis	[86]

Abbreviations used: ADAMTS-4, a disintegrin and metalloproteinase with thrombospondin motifs-4; ARDS, acute respiratory distress syndrome; BM, bone marrow; ECM, extracellular matrix; HIV-1, human immunodeficiency virus-1; HS, heparan sulfate; IGF, insulin-like growth factor; IGFBP-5, insulin-like growth factor binding protein-5; IL-1, interleukin-1; iNOS, inducible nitric oxide synthase; LPS, lipopolysaccharide; MSC, mesenchymal stem cell; NGF, nerve growth factor; OA, osteoarthritis; PPS, pentosan polysulfate; RA, rheumatoid arthritis; Tat, trans-activator of transcription; TIMP-3, tissue inhibitor of metalloproteinase-3.

**Table 2:** Inhibition of viral attachment to host cells, viral infection and replication by sulfated polysaccharides.

Virus	Inhibitor in Order of Potency	Study Type	References
African swine fever virus	λ-carrageenan,	IVL	[71]
	PPS,		
	κ-carrageenan,		
	Fucoidin		
Bovine viral diarrhea virus	PPS,	IVL	[252]
	Fucoidin,		
	Suramin,		
	Heparin,		
	Dermatan sulfate		
Coxsackievirus B3	PPS,	IVL	[253]
	Heparin		
Coxsackievirus A16	Heparin,	IVL	[255]
	PPS		
Dengue virus	Heparin,	IVL	[223]
	PPS,		
	Suramin,		
	PI-88		
Enterovirus 71	Heparin,	IVL	[255]
	PPS		
HIN1 influenza virus,	PPS,	IVL	[260]
H3N2 influenza virus	Dextran sulfate		
Herpes simplex virus-1, Herpes simplex virus-2	Dextran sulfate,	IVL	[226,227,228]
	PI-88,		
	Heparin,		
	PPS		
Herpes simplex virus, Human immunodeficiency virus-1, Vesicular stomatitis virus, Human cytomegalovirus	Dextran sulfate,	IVL	[225]
	λ-carrageenan,		
	PPS,		
	Fucoidin,		
	κ-carrageenan,		
	Heparin,		
Human cytomegalovirus	Dextran sulfate,	IVL	[226,244]
	PPS,		
	Heparin		
Human herpes virus 7	PPS,	IVL	[257]
	Dextran sulfate,		
	Heparin		
Human immunodeficiency virus-1	PPS,	IVL	[91]
	Dextran sulfate,		
	Heparin,		
	Fucoidin,		
	λ-carrageenan		
	κ-carrageenan,		
Human immunodeficiency virus-1	PS,	IVL	[229]
	Suamin,		[231]
	Hepa		[233]
	Dextran sulfate		[240]
Human immunodeficiency virus-1	Dextran sulfate,	IVL	[233]
	PPS,		[239]
	Heparin, Fucoidin		[240]
Human T-cell leukemia virus type-1	PPS	IVL	[74]
Japanese encephalitis virus	PPS,	IVL	[223]
	Heparin,		
	PI-88,		
	Suramin		
Junin virus, Tacaribe virus	Fucoidin,	IVL	[224]
	λ-carrageenan,		
	Dextran sulfate,		
	PPS,		
	Heparin		
Monkey pox virus	PPS	IVL	[281]
Sandfly fever Sicilian virus	Heparin,	IVL	[254]
	Suramin,		
	PPS,		
	κ-carrageenan,		
	λ-carrageenan,		
	Dextran sulfate,		
	Fucoidin		
SARS-CoV-2	PPS,	IVL	[7,24,264,265]
	Heparin		
Visna virus	PPS	IVL	[269]
Chikungunya virus	PPS	PCL	[271]
Ross River virus, Chikungunya virus	PPS	PCL	[72]
Human T cell leukemia virus type-1	PPS	PCL	[72]
AIDS-Karposi’s sarcoma Phase I CT	PPS	CT	[75]
AIDS-Karposi’s sarcoma Phase II CT	PPS	CT	[92]
Human T-cell leukemia virus type-1	PPS	CT	[268]
Ross River Virus Induced Arthralgia Phase IIa	PPS	CT	[73]

Abbreviations: CT, clinical trial; IVL, in vitro laboratory study; PCL, pre-clinical study.

**Table 3:** Established and potential new areas of therapeutic application for PPS.

1. Sports medicine treatment of damaged joint tissues including tendons, menisci, articular cartilage.
2. Promotion of tissue repair and regeneration and pain relief in OA/RA.
3. Promotion of IVD repair and regeneration and alleviation of low back pain.
4. Regulation of complement activation.
5. Modulation of vascular coagulation, fibrinolysis and thrombocytopenia.
6. Stimulation of HA synthesis by synoviocytes, fibroblasts.
7. Inhibition of NGF production by osteocytes providing pain relief in OA/RA.
8. Prevention of lipid accumulation in blood vessels in atherosclerosis.
9. Anti-inflammation: regulation of cytokine and inflammatory mediator production.
10. Anti-viral prevention of host cell infection/viral replication.
11. Anti-bacterial agent.
12. Anti-tumor agent.
13. Promotion of stromal MSC differentiation/proliferation/expansion for repair biology applications.
14. Promotion of protease inhibitor efficiency in tissues offering greater protection from proteolysis.
15. Cardio-, neuro- and chondro-protection.
16. Anti-inflammation: inhibition of leucocyte trafficking in inflamed tissues
17. Alleviation of kidney nephrotoxicity.

## Data Availability

All data is reported in the cited publications in this study.

## Abbreviations

AIDS, acquired immunodeficiency syndrome; ADAM, a disintegrin and metalloproteinase; ADAMTS, a disintegrin and metalloproteinase with thrombospondin motifs; AGE, advanced glycation end product; ASFV, African swine fever virus; AP-1, activating protein-1; APC, activated protein C; ARDS, acute respiratory distress syndrome; AZT, Zidovidine; AT, anti-thrombin; BM, basement membrane; BVDV, bovine viral diarrhea virus; CD4, cluster of differentiation-4 T-cell receptor; CHIKV; Chikungunya virus; CS, chondroitin sulfate; CVA16, Coxsackievirus A16; DENV, dengue virus; DMOAD, disease modifying osteoarthritis drug; DS, dermatan sulfate; DOX, doxycycline; ECM, extracellular matrix; ED50, antiviral effective dose 50%; EPCR, endothelial protein C inhibitor; aFGF, acidic fibroblast growth factor; GAG, glycosaminoglycan; GMK, green monkey kidney; HA, hyaluronan; HCII, heparin co-factor II; HCMV, human Cytomegalovirus; HHV-7, human herpes virus type 7; HIF-1α, hypoxia-inducible factor 1-alpha; HIV-1, human immunodeficiency virus-1; HSV, herpes simplex virus; HS, heparan sulphate; HuR, human embryonic lethal abnormal vision-like protein; IGFBP, insulin growth factor binding protein; IGF, insulin growth factor; IL, interleukin; iNOS, inducible nitric oxide synthase; IFN-γ, interferon-gamma; IVD, intervertebral disc; IVDD, intervertebral disc degeneration; JEV, Japanese encephalitis virus; JUNV, Junin virus; c-Jun, transcription factor Jun; KSHIV, Kaposi sarcoma human immunosufficiency virus; K-FGF, Kaposi’s sarcoma-derived fibroblast growth factor; LDL, low density lipoprotein; LPS, lipopolysaccharide; LMWH, low molecular weight HS; MCP, macrophage chemoattractant protein; MK, midkine; MMP, matrix metalloprotease; MPS, mucopolysaccharidoses; MSC, mesenchymal stem cell; NF-κB, nuclear factor kappa-light-chain-enhancer of activated B cells; NGF, nerve growth factor; NK, natural killer; NOD-SCID, nonobese diabetic/severe combined immunodeficiency; OA, osteoarthritis; PBMC, peripheral blood mononuclear cell; PCI, protein C inhibitor; PDGF, platelet derived growth factor; PEG, polyethylene glycol; PG, proteoglycan; PI3K/AKT, phosphoinositide 3-kinase protein kinase B; P38 MAPK, P38 mitogen-activated protein kinase; PTN, pleiotropin; RA, rheumatoid arthritis; RRV, Ross River virus; SARS-CoV-2, severe acute respiratory syndrome coronavirus-2; SFSV, sandfly fever Sicilian virus; TACV, Tacaribe virus; TAFI, thrombin activatable fibrinolysis inhibitor; TFPI, tissue factor pathway inhibitor; TGF-β, transforming growth factor beta; TIMP-3, tissue inhibitor of metalloproteases-3; TLR, Toll-like receptor; TNF-*α*, tumor necrosis factor-alpha; TPA, Tris(2-pyridylmethyl)amine; VSMCs, vascular smooth muscle cells; VCAM, Vascular cell adhesion molecule-1.
